# Modified Polycaprolactone Films for Temporary Protection in Saline Conditions: A Preliminary Assessment

**DOI:** 10.3390/polym18010060

**Published:** 2025-12-25

**Authors:** Am Pris John, Sergio Santoro, Efrem Curcio, Pietro Argurio, Francesco Chidichimo, Salvatore Straface, Silvestro Antonio Ruffolo, Mauro Francesco La Russa

**Affiliations:** 1 Department of Environmental Engineering (DIAm), University of Calabria, Via Pietro Bucci, 42B, 87036 Rende, Italy; sergio.santoro@unical.it (S.S.); efrem.curcio@unical.it (E.C.); pietro.argurio@unical.it (P.A.); francesco.chidichimo@unical.it (F.C.); salvatore.straface@unical.it (S.S.); 2 Department of Biology, Ecology and Earth Sciences (DiBEST), University of Calabria, Via Pietro Bucci, 12B, 87036 Rende, Italy; silvestro.ruffolo@unical.it (S.A.R.); mauro.larussa@unical.it (M.F.L.R.)

**Keywords:** biodegradable polymers, polycaprolactone (PCL), graphene oxide (GO), temporary barriers, archaeological conservation, seawater degradation, preliminary screening, laboratory incubation, provisional protection

## Abstract

Saline archaeological artifacts are highly susceptible to deterioration caused by salt crystallization and moisture–material interactions, particularly in coastal archaeological contexts affected by saline water intrusion. This persistent challenge necessitates the development of temporary, low-impact protective materials capable of limiting saline ingress. The present study reports on a preliminary assessment of modified polycaprolactone (PCL) films containing graphene oxide (GO) at 0.1%, 0.25%, and 0.5% to evaluate their potential as temporary barrier layers under saline stress conditions. Free-standing PCL/GO films were fabricated via solvent casting and exposed to natural Ionian seawater in a controlled laboratory incubation environment at 15 °C for up to 90 days, simulating early-stage saline exposure while controlling environmental variability and physical stress. Film behavior was evaluated through complementary surface, structural, mechanical, and permeability analyses. The findings indicate that GO content significantly influences surface wettability, microstructural evolution, and water transport properties. Low GO content (0.1%) enhanced barrier performance while maintaining structural integrity and controlled hydrolytic softening. In contrast, higher GO contents (0.25–0.5%) resulted in increased hydrophilicity, accelerated surface erosion, and greater mechanical degradation due to enhanced water uptake. Observed mass loss is attributed to early-stage hydrolysis rather than long-term biodegradation. This investigation is a material-level screening and does not represent a direct validation for conservation application. With superior stability and enhanced barrier properties, the optimized PCL/GO 0.1% film suggests significant potential for the protection of saline-affected archaeological materials.

## 1. Introduction

Archaeological materials discovered in saline conditions are susceptible to complex and rapid deterioration, including salt crystallization, ion exchange, and chloride-related corrosion. Such cycles of salt dissolution and recrystallization, enhanced by changing humidity and temperature, introduce critical crystallization pressures within porous substrates such as stones, mortar, and earthen materials, leading to microfracturing, delamination, granular disintegration, and surface powdering [1,2,3,4,5,6,7,8,9]. Then, capillary rise introduces chloride-, sulphate- and nitrate-rich brines into calcareous materials, where phases such as magnesium sulphate and gypsum cause additional structural damage [3,4]. Chloride infiltration in metals causes electrochemical corrosion, generating voluminous corrosion products that significantly hinder effective desalination and long-term conservation [9,10,11,12].

Although archaeological artifacts may remain relatively stable during long-term burial in saline or marine environments, deterioration frequently accelerates following excavation due to abrupt environmental changes. Exposure to oxygen, humidity fluctuations, and drying can activate salt migration and crystallization, while residual chlorides may induce corrosion or mechanical stress. Consequently, the post-recovery phase represents a critical period in which temporary stabilization measures may be required prior to desalination and long-term conservation treatments [13].

Recent conservation research has shifted, focusing on the development of temporary and low-impact materials that are reversible and suitable for archaeological site preservation and post-excavation stabilization [14,15,16,17,18]. Biodegradable polymers have emerged as promising candidates due to their low environmental footprint, tunable degradation behavior, and favorable mechanical and barrier properties. Among them, polycaprolactone (PCL) has gained wide attention in biomedical fields, packaging, and agriculture where its performance can be enhanced through nanocomposite formation, polymer blending, or chemical modification [19,20,21,22,23]. When PCL is used in conjunction with modified cellulose, starch, or polylactic acid, the strength of composite materials, flexibility, and degradation rate change. Nanofillers such as reduced graphene oxide or lignin nanoparticles provide enhanced antimicrobial properties, increased resistance to ultraviolet radiation, and improved moisture protection [24,25,26,27,28]. Importantly, the degradation rate of PCL can be tailored by modifying filler loading and microstructural design, enabling the functional lifespan of PCL products to be individually tailored for short-term conservation applications [28,29,30,31,32,33].

Graphene oxide (GO) is recognized as a multifunctional nanofiller due to its high surface area, abundant oxygen-containing functional groups, and strong interactions with polymer matrices [34]. Significantly, incorporation of GO at low concentrations has been shown to enhance the mechanical properties, stiffness, and structural stability of polymer matrices [32,33,34,35,36]. When GO content is at or below 0.1 wt%, the GO sheets disperse uniformly throughout the PCL matrix. In this optimal state, GO acts to increase the tortuosity of the polymer, introducing elongated diffusion pathways for water molecules that significantly diminish the permeability of the PCL film, thereby creating an enhanced barrier [37,38,39]. However, water transport behavior changes when the GO content exceeds 0.5 wt%. At these higher loadings, the composite is dominated by the GO’s surface chemistry and poor dispersion [40,41]. Similar trends in other polyester–GO systems report that higher GO loadings increase water absorption, which may reduce barrier performance [42,43]. Besides, GO contents above 0.5 wt% often promoted agglomeration and surface irregularities, resulting in non-uniform film morphology that is unsuitable for heritage conservation applications [44,45]. Therefore, a GO range of 0.1–0.5 wt% was selected to balance dispersion quality, mechanical flexibility, and barrier performance while avoiding agglomeration and brittleness at higher loadings [46].

The hydrophobicity and water-impermeability imparted by GO can be quantified by measuring contact angle changes in various polymer systems, including polyurethanes, epoxies, and biopolymers [47]. In addition, functionalization groups, such as amine, silane, or isocyanate, improve compatibility and prevent GO agglomeration, resulting in a consistent enhancement of barrier performance [34,48,49].

Studies on PCL and PCL-based nanocomposites in saline or marine environments consistently demonstrate slow degradation kinetics and a strong dependence on temperature, crystallinity, and filler content [50]. Typically, PCL exhibits mass loss in natural seawater over several weeks due to limited diffusion of water into its semi-crystalline regions and low microbial activity at lower temperatures [51,52]. While low concentrations of GO can improve performance by advancing PCL crystallinity and reducing water permeability, higher loadings often tend to introduce hydrophilic groups, leading to faster hydrolysis [53]. Many findings demonstrated the occurrence of surface erosion and morphological degradation in PCL-based composite materials when tested in both marine and hydrolytic environments [54,55,56,57]. Despite existing findings, the fundamental link between PCL/GO microstructural evolution in seawater and its practical suitability as a temporary protective barrier for marine archaeological objects remains systematically unexamined [14].

In conservation practice, temporary protective materials must comply with key principles, including reversibility, chemical compatibility, and minimization of risk to the artifact [58,59]. The principle of reversibility is particularly critical, requiring that barrier films leave no residue and cause no chemical or physical change to the underlying substrate upon removal [60]. Hence, the interactions between the polymer films and soluble salts are highly significant for archaeological materials because of the effect of saline environments, which can impact the performance of the polymers by swelling, accelerating hydrolysis, and affecting surface stability [61,62]. When incorporating nanomaterials such as GO, potential risks related to particle transfer, surface contamination, or changes in substrate appearance must be carefully evaluated [63,64].

In cultural heritage conservation, biodegradable materials must be applied with caution. While biodegradability offers environmental advantages, microbial activity on artifact surfaces can pose risks, including biofilm formation and surface alteration [65]. In the present study, biodegradability is considered only as an end-of-life property of the material and not intended as a functional mechanism for artifact protection. Potential microbial and substrate-interaction risks are acknowledged and reserved for future investigations.

Accordingly, this work focuses on the preliminary material screening of free-standing PCL/GO films under saline conditions. This assessment aims to assess surface behaviour, internal microstructure, and functional performance in order to identify formulations that are suitable for temporary stabilization. We acknowledge, however, that further investigations are required to evaluate conservation parameters such as adhesion, removability, chemical stability, and safe application on archaeological artifacts.

## 2. Materials and Methods

### 2.1. Materials

**Figure 1:**
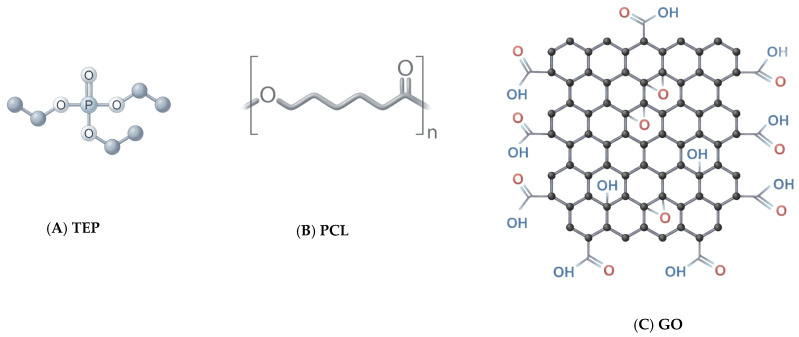
Chemical structures of (**A**) triethyl phosphate (TEP), (**B**) polycaprolactone (PCL), and (**C**) graphene oxide (GO).

For preparation, polycaprolactone with an average molecular weight of (PCL, Mn ≈ 80 kDa; Cat. No. 796034), Graphene Oxide nanopowder (GO, 15–20 sheets, 4–10% edge-oxidized, average number of layers, 15–20; Cat. No. 1003059893), and triethyl phosphate (TEP, ≥99%; Cat. No. 102252228) were all purchased from Sigma-Aldrich (St. Louis, MO, USA) and utilized without further purification. Chemical structures of these materials are illustrated in Figure 1. In March 2024, natural seawater samples from the Ionian Sea were collected at Crotone (Calabria, Italy). The water temperature at the time of collection was 15 °C.

### 2.2. Preparation of PCl/GO Composite Films

**Figure 2:**
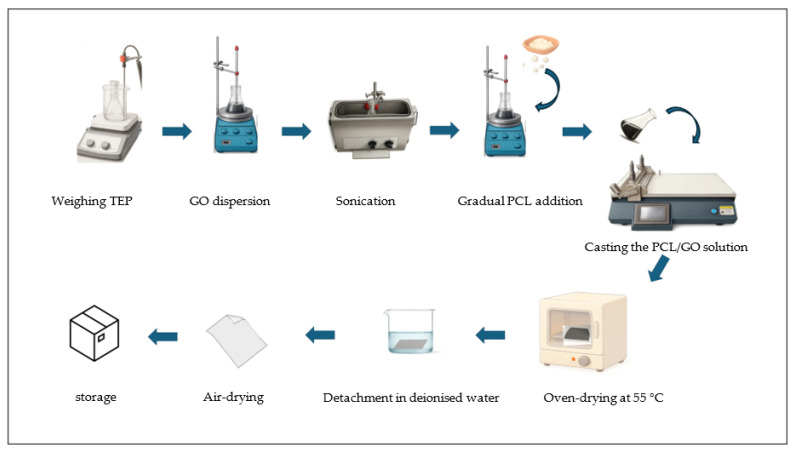
Schematic workflow of the preparation of PCL/GO films, detailing the steps from component dispersion to final film formation.

Composite Films of PCL/GO were prepared with varying amounts of graphene oxide (0.1 wt%, 0.25 wt%, or 0.5 wt% GO). See Figure 2. Initially, TEP was weighed as a green solvent and placed in a glass beaker, then GO nanopowder was added to TEP and mixed using a magnetic stirrer to achieve an initial dispersion. Then, the mixed solution was sonicated for 30 min using a VWR Ultrasonic Cleaner (VWR International, Radnor, PA, USA). Pellets of PCL were gradually added to the solution dropwise in three portions, while stirring was maintained at 55 °C as a preparation temperature. Following this mixing period, the solution was heated for an additional 3 h, which was sufficient time for the polymer to fully dissolve.

In this time frame, we included an additional 2 h for the solution to rest undisturbed, allowing any gases or bubbles that formed during the mixing process to escape and creating a more homogeneous final solution. The resulting viscous, homogeneous polymer suspension was deposited onto a clean glass plate using a doctor blade. The resulting films were dried in a drying oven at 55 °C for 12 h. This step enabled controlled evaporation and limited the temperature to the melting point of PCL. Upon completion, the glass plate was submerged in deionized water and subsequently released from its substrate. The resulting films were air-dried at room temperature for 24 h prior to being characterized. Notably, residual TEP from the solvent-casting process may remain in the films and function as a mild plasticizer, thereby influencing mechanical behavior. Conversely, identical fabrication conditions ensure comparable residual solvent content across tested PCL/GO formulations. The thickness of the films was measured at multiple locations on each film using a digital micrometer, with values ranging from 0.1 mm to 0.22 mm, respectively.

The behavior of pure PCL under comparable solvent-casting and saline immersion conditions has been reported previously [14] and is used here as the contextual reference for interpreting trends observed in the GO-modified films. Accordingly, the present study focuses on comparative material screening of PCL/GO films, rather than re-establishing the full baseline behavior of PCL.

### 2.3. Chemical Composition of Ionian Seawater Used in Immersion Tests

Ionian Seawater taken from Crotone (Italy) was analyzed prior to testing its permeability and biodegradability. The main cations (Na^+^, K^+^, Mg^2+^, and Ca^2+^) were identified and quantified using the Atomic Absorption Spectrophotometer (Analytik Jena Contra AA 700, Jena, Thuringia, Germany). The major anions (Cl^−^ and SO_4_^2−^) were analyzed using an Ion Chromatograph (Metrohm 930 Compact IC Flex, Herisau, Switzerland). A calibrated pH meter (Hanna Instruments HI2211, Limena, Italy) was used for the measurement of the pH of the seawater.

To approximate the saline conditions experienced by buried coastal artifacts, PCL/GO films were immersed in natural Ionian seawater, which was selected due to its representative salinity and relevance to the coastal archaeological context of Crotone (Italy). A controlled temperature of 15 °C was selected, matching the temperature of the collected field seawater, in order to minimize microbial activity throughout the preliminary material assessment.

The seawater immersion tests were not intended to simulate a museum display or permanent underwater exhibition. Instead, seawater was used as a controlled saline stress environment to preliminarily screen the behavior of free-standing PCL/GO films, including water transport and early-stage hydrolytic responses.

### 2.4. Surface Behavior of the Films

#### 2.4.1. Surface Hydrophobicity: Contact Angle Measurements

The sessile drop method was applied to determine how readily each surface could be wetted by water. To minimize gravitational spreading and edge effects on thin polymer films, a 2-µL droplet of deionized water was added. Measurements were then performed at three separate locations on each sample, and the results were subsequently averaged to reduce variability associated with small droplet volumes. The three contact angle values for each film were averaged to provide a single value for each film.

#### 2.4.2. FTIR Spectroscopy

Fourier Transform Infrared (FTIR) spectra from FTIR spectroscopy instruments using an ATR crystal attachment over the spectral range of 500–4000 cm^−1^ and taking 32 scans on a per-sample basis at 4 cm ^−1^ resolution. FTIR analysis was used to characterize the chemical groups present in the films and determine the chemical stability of each film sample.

### 2.5. Internal Microstructure and Film Quality

#### SEM Imaging

Scanning electron microscopy (SEM) was used by researchers to analyze films containing GO, and to define GO morphology, pore structure, and arrangement. All samples were coated with a very thin layer of gold via sputter coating. Researchers acquired SEM images of the film samples at multiple accelerating voltages from 5 through 15 kV.

### 2.6. Functional Protection Performance

#### 2.6.1. Permeability Testing

**Figure 3:**
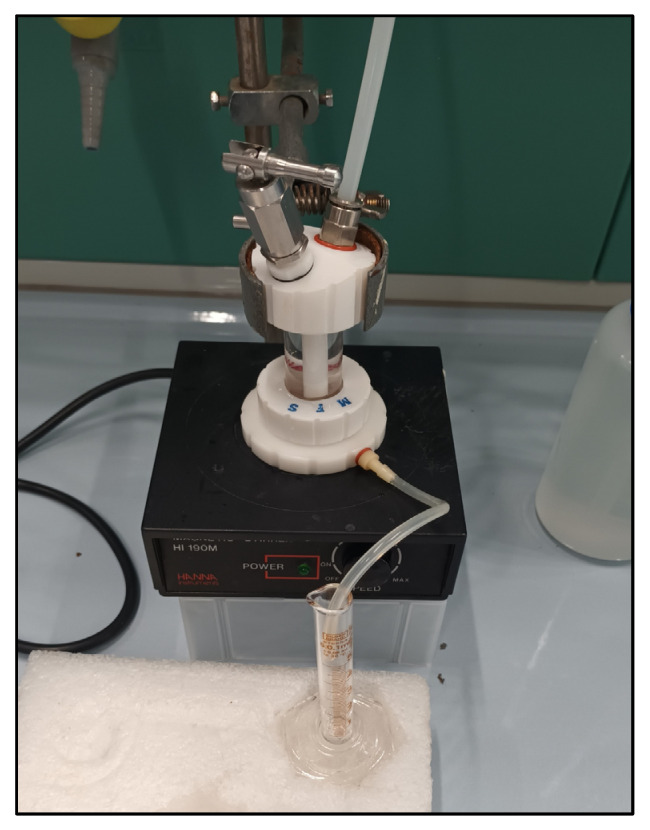
Permeability testing utilized the UHP-25 cell (top), where the film sample was mounted and sealed with an O-ring to ensure controlled flow, and permeate is collected (bottom) under constant pressure for calculating flux and permeability.

The permeability can determine how effectively a film prevents saline water from penetrating archaeological artifacts. Therefore, seawater permeability testing is a valuable method for estimating the potential for saline erosion or damage to artifacts stabilized under the film. As shown in Figure 3, the seawater permeability testing of the films evaluated in this study was performed using a dead-end filtration cell (UHP-25, Strelitech, Osaka, Japan) operated at 0.5 bar feed pressure and a magnetic stir plate maintaining a constant stirring speed (190 rpm) at room temperature.

Circular film test strips having an effective area of 19.6 cm^2^ were placed between the feed (treated seawater) and permeation chambers. The weight loss of the film samples resulting from magnetic stirring of the film, and the increase in conductivity readings measured in the chamber over the next 6 h of testing, were recorded at time intervals. The higher the permeability of the tested films, the greater the possibility of saline erosion of archaeological artifacts. The permeate flux and permeability of the films were used in these two equations:

(1) $$\;J\;=\frac{Q}{A\cdot t}$$

where *J*: permeate flux (L/m^2^·h); *Q*: volume of permeate collected (L); *A*: membrane area (m^2^); *t*: time (h).

(2) $$P=\frac{J}{∆P}$$

where *P*: permeability (L/m^2^·h·bar); *J*: permeate flux (L/m^2^·h); Δ*P*: applied pressure difference (bar).

#### 2.6.2. Mechanical Testing

**Figure 4:**
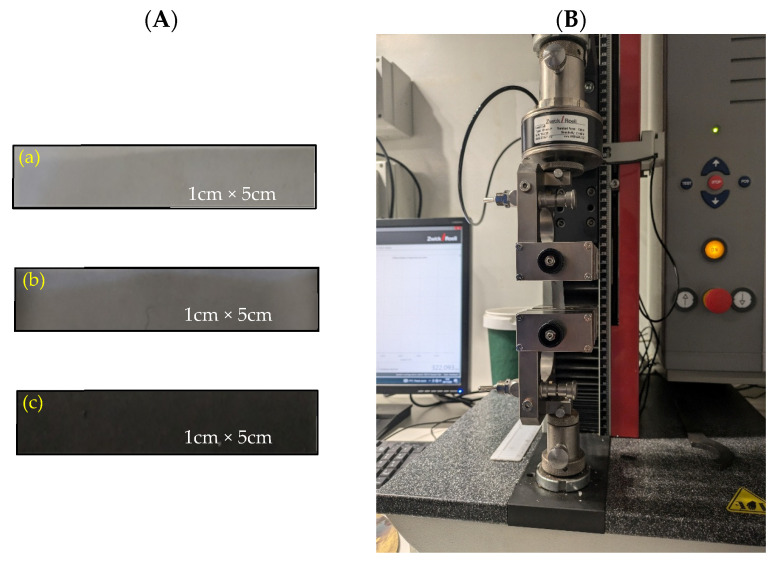
Tensile testing setup and specimens used for uniaxial tensile measurements: (**A**) representative PCL/GO film strips, including (typical dimensions: 1 cm × 5 cm), including (**a**) PCL/GO 0.1 film, (**b**) PCL/GO 0.25 film, and (**c**) PCL/GO 0.5; (**B**) the universal testing machine (UTM) used for mechanical characterization.

The mechanical properties of GO films were assessed using Zwick/Roell Z2.5 Universal Test Equipment (BTC-FR2.5TN-D09, Zwick Roell Group, Ulm, Germany). As illustrated in Figure 4, the strips of film evaluated were rectangular (1 cm wide, 5 cm long) in triplicate. All tests were performed on samples in uniaxial tensile mode at a constant crosshead speed of 5 mm/min until rupture. Each film was tested with three strips to determine the average Young’s Modulus (MPa) and elongation at break (%) for the strips included in each film formulation.

### 2.7. Long-Term Stability in Saline Environments

#### Hydrolytic Mass Loss Testing

**Figure 5:**
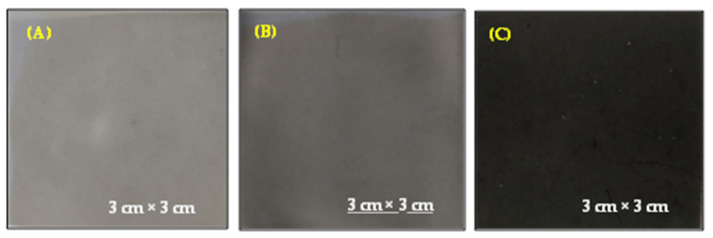
Physical appearances of the films of (**A**) PCL/GO 0.1 film, (**B**) PCL/GO 0.25 film, and (**C**) PCL/GO 0.5 cut to 3 cm and 3 cm dimensions.

PCL/GO films were determined based on their % Weight Loss after immersion in seawater for 90 days; these physical images are presented in Figure 5. Three square films were dried to constant weight, immersed, and weighed relative to one another. The weights of the films were altered every 30 days and air-dried before being weighed again. All weights were taken based on the drying results. Mass loss was calculated as follows:

(3) $$Weight\;loss\;(\%)=\frac{W_{0}-W_{t}}{W_{0}}\;\times 100$$

where *W*_0_: the initial weight of the sample before biodegradation (g); *W_t_*: the weight of the sample after biodegradation (g).

## 3. Results and Discussion

Based on the combined experimental results, the behavior of the modified GO films is interpreted relative to literature-reported PCL behavior under comparable saline conditions. Observed variations among the composite films correlate with GO concentration.

### 3.1. Seawater Characterization

The ionic composition of seawater collected along the Ionian coastline of Crotone can be found in Table 1. The primary anion and cation are Cl^−^ and Na^+^, respectively, which are two of the main components of seawater’s salinity. Moreover, there are significant concentrations of other important ions present in seawater, such as Mg^2+^, Ca^2+^, K^+^, and SO_4_^2−^. Therefore, seawater provides sufficient dissolved ions for the study of polymer degradation under marine conditions. In addition, particular importance is given to Mg^2+^ and Ca^2+^ concentrations, along with the relatively high chloride concentration, because these ions can affect the rate of hydrolysis, the stability of the polymer surface, and the degradation of polymers such as PCL.

### 3.2. Surface Behavior of the Films

#### 3.2.1. Surface Hydrophobicity: Contact Angle Measurements

**Figure 6:**
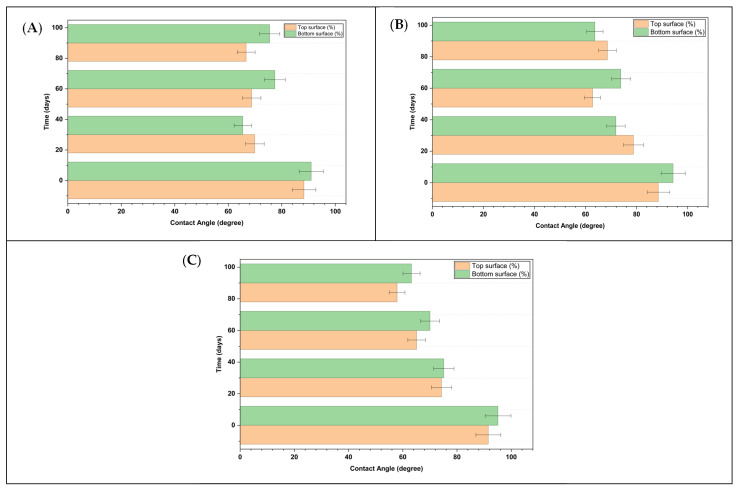
Surface wettability (contact angle) of PCL/GO composite films. Measurements are illustrated for the bottom (upper bars) and top surfaces (lower bars) of (**A**) PCL/GO 0.1 film, (**B**) PCL/GO 0.25 film, and (**C**) PCL/GO 0.5 film, highlighting the influence of GO concentration.

The contact-angle measurements showed reproducible trends across multiple locations and replicates, successfully mitigating the experimental variability associated with reduced droplet volumes. As indicated in Figure 6, the PCL/GO films showed a gradual decrease in surface hydrophobicity due to the gradual 90-day exposure to seawater. All measurements are reported as mean ± standard deviation (*n* = 3), and error bars in the figures represent the corresponding standard deviation.

Differences between the top and bottom surfaces are attributed to the doctor-blade casting process, caused by blade-induced shear and faster solvent evaporation at the air-exposed surface. On day 0, all films showed contact angles above 80°, indicating moderate hydrophobicity, although the bottom surfaces were more hydrophobic than the top surfaces due to differences in casting morphology.

After 30 days of seawater immersion, a significant reduction in contact angle was observed across all GO concentrations, demonstrating that wetting and hydrolytic interactions between the bulk chemical structure of the films and seawater occur during the early phase of contact. By 60 and 90 days, contact angles continued to decrease, particularly for the 0.25% and 0.5% GO films. The observed decrease in contact angle is likely influenced by surface roughening effects, consistent with the Wenzel wetting regime, rather than exclusively attributed to changes in surface chemistry. This condition may reflect the combined effects of surface roughening and microstructural changes.

Overall, the 0.1% GO film displayed lower surface wettability compared to the higher GO content samples.

#### 3.2.2. FTIR Spectroscopy

**Figure 7:**
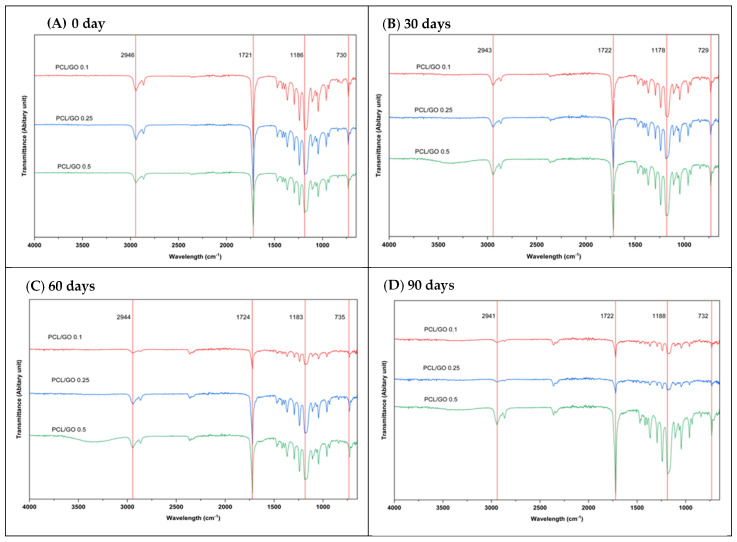
FTIR spectra for PCL/GO films for times of immersion into natural seawater of (**A**) 0, (**B**) 30, (**C**) 60, and (**D**) 90 days, indicating changes occurring to bulk functional groups of the films and the general chemical integrity.

The FTIR spectroscopy for the PCL/GO polymers prior to, and on Days 30, 60, and 90 post seawater immersion, is illustrated in Figure 7. The PCL/GO polymers on Day 0 of Figure 7A display the PCL characteristic peaks and bands with 1721–1724 cm^−1^ carbonyl stretch, 2943–2946 cm^−1^ CH_2_ stretching, 1183–1188 cm^−1^ C-O-C bands, and 729–735 cm^−1^ crystalline bands.

The spectra variations at 30 days and 60 days of Figure 7B,C were nearly identical to each other except for a slight reduction in intensity of the carbonyl and C-O-C bands. Additionally, the spectrum broadened around the CH_2_ stretching region. The slight shifts in the carbonyl and C-O-C bands and the broadening of the spectrum are associated with minimal mass loss at these time points due to the initiation of hydrolysis, although the process did not reach its maximum extent.

Spectral variations occurred in the films after 90 days, as shown in Figure 7D, with a corresponding decrease in intensity for both the carbonyl and ether (C-O-C) bands as a consequence of hydrolytic degradation of PCL/GO films due to early breakdown of the ester bonds. There were no significant shifts in FTIR peak positions observed, and variations in peak intensity were identified. These intensity changes are discussed qualitatively, as spectra were not normalized.

Studies on PCL/GO composites demonstrated that low GO loadings (0.1–0.5 wt%) do not significantly alter the backbone chemistry of PCL, as shown by unchanged FTIR spectra prior to degradation and hydrolysis [66]. These results support that the intensity reduction observed in our study at 90 days during seawater exposure was mainly caused by hydrolytic chain scission, not oxidative degradation.

### 3.3. Internal Microstructure and Film Quality

#### SEM Imaging

**Figure 8:**
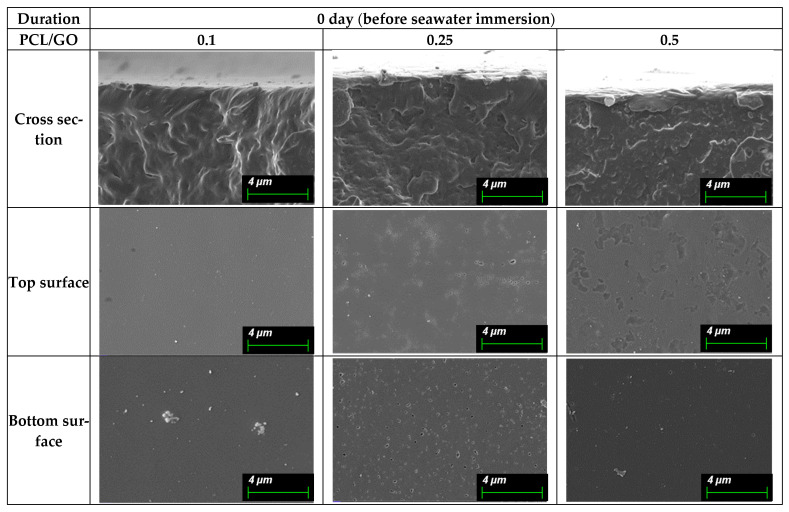
SEM micrographs of PCL/GO films with different GO concentrations (0.1%, 0.25%, and 0.5%) at day 0 (before seawater immersion), showing smooth and compact surface morphology and intact cross-sectional structure. Micrographs were captured at the 4 µm scale highlight finer surface features and confirm the absence of pits, cracks, or degradation-related defects prior to exposure.

**Figure 9:**
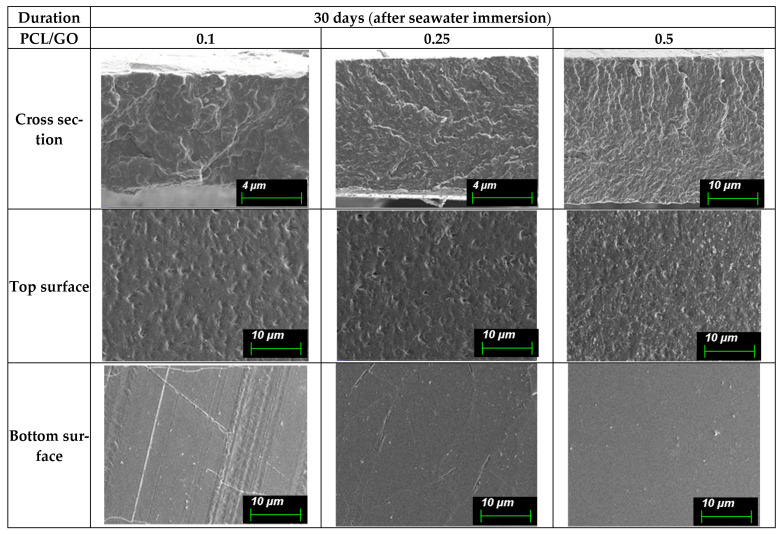
SEM micrographs of PCL/GO films with different GO concentrations (0.1%, 0.25%, and 0.5%) after 30 days of seawater immersion. The micrographs at 10 µm scale provide an overview of surface morphology and onset of surface interaction with saline environment, while 4 µm scale images highlight finer surface features associated with early stage roughening and initial appearance of small pits.

**Figure 10:**
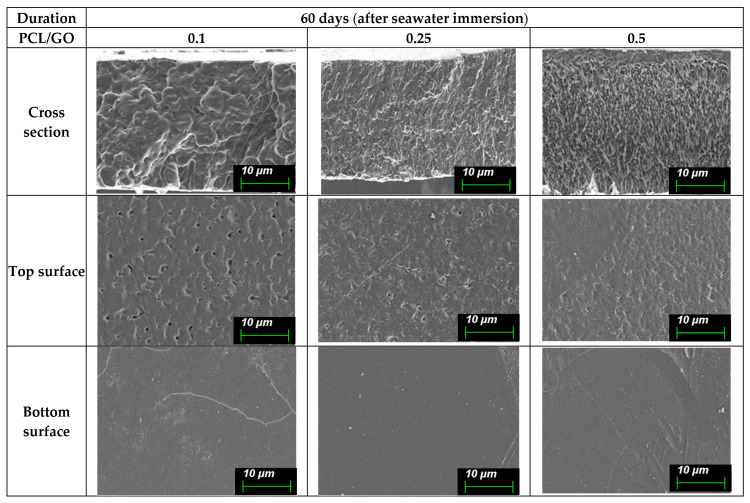
SEM micrographs of PCL/GO films with different GO concentrations (0.1%, 0.25%, and 0.5%) after 60 days of seawater immersion, displaying increased surface degradation, including microcracks and enhanced roughness. Images acquired at a 10 µm scale provide an overview of surface morphology and pit distribution associated with prolonged saline exposure.

**Figure 11:**
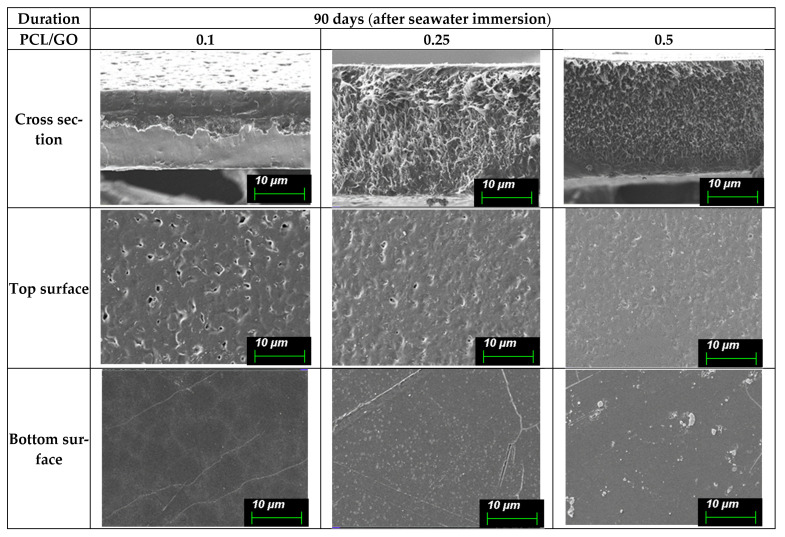
SEM micrographs of PCL/GO films with different GO concentrations (0.1%, 0.25%, and 0.5%) after 90 days of seawater immersion, showing advanced deterioration with extensive cracking, roughening, and structural disruption, with severity increasing at higher GO loadings. Micrographs acquired at a 10 µm scale provide an overview of large-scale features and pit distribution associated with prolonged saline exposure.

Since the observed SEM surface features of the films may reflect both early-stage hydrolytic effects and mechanical artifacts presented during sample preparation, morphological changes are discussed qualitatively, avoiding their use as direct measures of material degradation. These surface changes are shown in (A) Figure 8 (0 day), (B) Figure 9 (30 days), (C) Figure 10 (60 days), and (D) Figure 11 (90 days), comparing film morphology before and after seawater exposure. Different SEM magnifications (10 µm and 4 µm) were intentionally applied to capture both surface morphology and finer degradation-related features. The lower magnification (10 µm) provides an overview of pit distribution, while the higher magnification (4 µm) allows for a detailed observation of early-stage roughening and microstructural changes.

On day 0, all PCL/GO films exhibit dense and continuous cross-sectional structures, confirming that the solvent-casting method produced compact, defect-free films. See Figure 8. The PCL/GO 0.1% film shows a relatively uniform lamellar arrangement, while the 0.25% and 0.5% films display slightly more irregular or textured cross-sections, suggesting that higher GO loading may introduce mild microstructural variations.

On the surface, the films appear smooth and homogeneous. The top surfaces present a clean, featureless morphology, while the bottom surfaces are even smoother, reflecting good leveling during film formation. No visible cracks, pores, or particle agglomerates are observed, indicating good film quality before seawater exposure.

Overall, the films demonstrated robust structural integrity prior to seawater exposure, providing a consistent baseline for subsequent immersion studies.

After 30 days of seawater immersion, the cross-sectional structures remain dense with no major voids, although mild roughening becomes visible at the polymer–air interface. See Figure 9. The top surfaces of all samples show early signs of microtexturing, giving a slightly rougher appearance than day 0. These subtle morphological changes indicate the onset of surface interaction with seawater.

The PCL/GO 0.25% and 0.5% films show more noticeable surface irregularities than the PCL/GO 0.1% films, with finer pits or shallow features beginning to appear. Bottom surfaces remain mostly smooth, with only minor scratches or irregular lines that are likely related to handling rather than degradation.

In general, SEM observations indicate that seawater exposure primarily induces progressive surface-level morphological changes, while the bulk structure of the PCL/GO films remains largely intact over the study period. The extent of surface roughening advanced proportionally with immersion time and GO content, consistent with enhanced water–film interactions under saline conditions.

At 60 days, morphological changes in Figure 10 become more evident. Cross-sections show increased internal roughness, and faint porous regions or micro-channels appear within the film thickness. These features suggest that seawater has penetrated deeper into the polymer matrix.

The top surfaces now show clear pitting and roughening, especially in the 0.25% and 0.5% films. These small pits and depressions indicate ongoing surface erosion. The 0.1% GO film shows fewer and smaller pits, retaining a more compact surface texture.

Bottom surfaces exhibit visible cracks or long, shallow lines in some samples. These features are more distinct in films with higher GO loading, indicating that structural stress or swelling may be occurring unevenly.

Collectively, it is noted that the degradation severity is directly proportional to the GO loading after 60 days.

The degradation patterns shown in Figure 11 are significantly more evident after 90 days. Cross-sections show substantial internal roughening and the presence of larger pores or fractured regions, particularly in the 0.25% and 0.5% films. These indicate progressive hydrolysis and erosion within the film thickness.

Top surfaces display widespread pitting and deeper cavities, giving a clearly degraded appearance. The 0.1% film, although affected, still shows fewer defects than the higher GO samples.

Bottom surfaces exhibit larger cracks, fragmented patterns, or irregular breakage lines, especially in the 0.5% film. This suggests that prolonged immersion leads to structural weakening and surface fracture, especially at higher GO contents.

Therefore, the long-term performance of 90 days limit of PCL/GO films in this environment. Only low GO concentrations may offer acceptable long-term stability, while higher concentrations are unsuitable for applications requiring durability beyond 60–90 days in saline conditions.

### 3.4. Functional Protection Performance

#### 3.4.1. Permeability Testing

**Figure 12:**
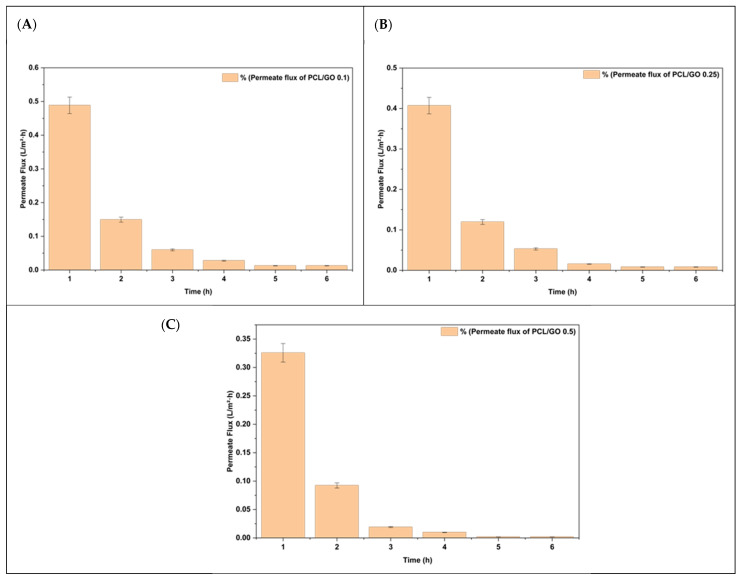
The permeate flux rates of PCL/GO films containing different graphene oxide concentrations as a function of exposure time: (**A**) 0.1 wt%, (**B**) 0.25 wt%, and (**C**) 0.5 wt%. Values are reported as mean ± standard deviation (*n* = 3).

We conducted a 6 h permeate flow assay for PCL/GO films using the UHP-25. The experimental results are demonstrated in Figure 12. For all three formulations, the flux exhibits a steep decline between 1 and 3 h, followed by a plateau approaching near-zero values at longer times. Its behavior reflects rapid initial wetting of the film surface, followed by the formation of a stable barrier layer that prevents further saltwater penetration.

As illustrated in Figure 12A, the PCL/GO 0.1% film demonstrates that the initial flux was 0.55 L·m^−2^·h^−1^. After 3 h, there was a rapid decline, reaching <0.05 L·m^−2^·h^−1^. The flux remained at that level for the rest of the test. This stabilization indicates the formation of a dense, water-saturated layer that hinders further permeation through the film.

The PCL/GO 0.25% film Figure 12B has a lower initial flux than the 0.1% sample (0.45 L·m^−2^·h^−1^) and stabilizes at an even lower level than the 0.1% sample after 3 h. The enhanced barrier effect observed with the higher GO content compared with the lower GO concentration is attributed to the tortuous pathways formed by the nanosheets due to the increased GO loading.

The PCL/GO 0.5% film, showing in Figure 12C, exhibited the lowest initial flux of the three samples (0.32–0.35 L·m^−2^·h^−1^), which quickly fell to near-zero levels within 2–3 h. While it had the lowest steady-state values, this observation can be attributed to the enhanced restrictions imposed by increased physical obstructions and reduced free volume that occurred as the GO loading level increased.

Higher GO content reduces initial flux and accelerates the transition to a low, steady-state regime. This indicates that GO improves the barrier properties of PCL films against saltwater diffusion, with the performance observed for the 0.5% GO formulation.

**Figure 13:**
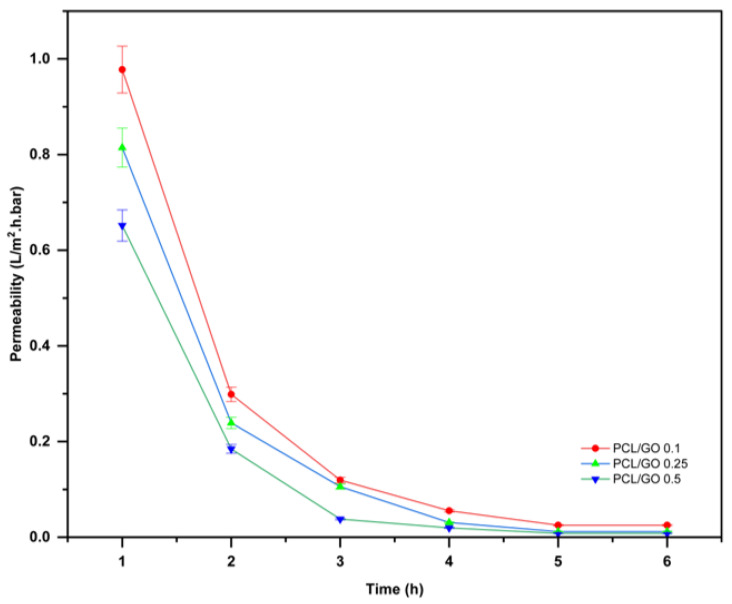
Permeability of PCL/GO films over a 6 h-filtration period, summarizing permeability values extracted from steady-state regions. Values are reported as mean ± standard deviation (*n* = 3).

Figure 13 presents the permeability coefficients of the PCL/GO films measured over a 6 h interval. In all films, the permeability decreases sharply during the first 2 h, followed by a gradual decline to near-zero values between 4 and 6 h. This behaviour reflects the rapid initial wetting and subsequent compaction of the polymer–GO structure under hydraulic pressure, leading to restricted saltwater transport.

Permeability measurements were performed on mechanically intact films. Any minor mass loss observed at later stages is attributed to handling during prolonged immersion and does not affect the validity of the permeability assessment.

At the initial time point, PCL/GO 0.1% film exhibited a permeability of approximately 0.95 L·m^−2^·h^−1^·bar^−1^. PCL/GO 0.25% and PCL/GO 0.5% films displayed much lower permeability (approximately 0.85 L·m^−2^·h^−1^·bar^−1^ and 0.65 L·m^−2^·h^−1^·bar^−1^, respectively) and were consistent with our prediction that films containing higher concentrations of PCL and GO would likely have higher probabilities of developing tortuous diffusion pathways and slow or impede water penetration through the film matrix. During the 1- to 2 h time interval, the permeability values dropped (~0.25–0.30 L·m^−2^·h^−1^·bar^−1^), and the data indicate that a saturated film structure is likely created at this time point along with the establishment of a limit of hydration.

Continuing to monitor the films at 3- to 6 h intervals, we observed overall convergence in permeability across all PCL/GO films to low (<0.05 L·m^−2^·h^−1^·bar^−1^) levels. PCL/GO 0.5% film demonstrated lower permeability values than other types throughout the testing, supporting our earlier premise that higher GO concentrations would improve the barrier performance of films by increasing resistance to diffusion. Conversely, PCL/GO 0.1% film consistently displayed slightly higher values for permeability during all evaluations, although it was ultimately negligible (<0.05 L·m^−2^·h^−1^·bar^−1^) at 4 h.

Overall, PCL/GO films can slow saltwater infiltration by significantly increasing permeability with increasing GO concentration. Therefore, our results support the hypothesis that increased GO concentrations can significantly reduce saltwater infiltration and permeability in polygraphene nanocomposite materials [67,68].

#### 3.4.2. Mechanical Testing

Table 2 shows the changes in Young’s modulus and elongation at break of the PCL/GO composite films during 90 days of immersion in natural Ionian seawater. At day 0, all films displayed comparable mechanical behavior, with Young’s modulus ranging from 284 to 331 MPa and elongation values between 5.7% and 7.7%. These values reflect the typical balance of stiffness and ductility observed in freshly prepared PCL-based nanocomposites [69,70]. Although the GO loadings were low (0.1–0.5 wt%), slight differences in the initial Young’s modulus value of the GO composite films suggest the GO platelets incorporated into the polymer matrix also contributed positively.

After 30 days of immersion, differences among the GO concentrations become more apparent. The PCL/GO 0.1% film exhibits a pronounced increase in stiffness (414 MPa), indicating structural densification or enhanced polymer chain ordering during initial seawater exposure. In contrast, the modulus of the 0.25 wt% film increased only slightly, while the 0.5 wt% film exhibited a substantial decrease (166 MPa), indicating greater sensitivity to water uptake at higher GO contents. In all samples, elongation at break decreases at 30 days (4–5%), demonstrating early-stage embrittlement associated with hydration-induced chain rearrangement.

By 60 days, all experienced a significant reduction in Young’s modulus, reaching 183, 163, and 153 MPa for the 0.1, 0.25, and 0.5 wt% GO films, respectively. This decline reflects the onset of hydrolytic degradation and progressive loss of structural integrity. Concurrently, elongation at break increased for the 0.25 wt% and 0.5 wt% films (8.9% and 8.0%), demonstrating increased chain mobility and partial softening of the polymer matrix.

After 90 days, mechanical degradation was evident for all samples, with Young’s modulus decreasing to 104–144 MPa, confirming advanced structural weakening. However, elongation at break increased sharply for the 0.1 wt% and 0.5 wt% films (19.7% and 20.7%), representing a transition toward more ductile behavior of advanced hydrolytic softening in polyester systems and partial plasticization, likely associated with water uptake and enhanced polymer chain mobility, rather than mechanical degradation. Moreover, the 0.25% film shows a more moderate increase (5.4%), suggesting a different degradation pattern that balances embrittlement and softening.

It should be noted that the presence of residual triethyl phosphate, originating from the solvent-casting process, may contribute to the observed softening behavior and increased elongation at break, particularly at longer immersion times. However, all films were prepared and dried under identical conditions. The comparative trends observed among different GO loadings primarily reflect the influence of graphene oxide rather than variations in residual solvent content. The observed mechanical softening is attributed to gradual seawater diffusion into the amorphous regions of the PCL/GO films, causing polymer plasticization without evidence of structural failure.

Among the formulations, the PCL/GO 0.1 wt% film demonstrates the greater mechanical stability over the exposure period, retaining higher modulus values and more controlled ductility changes, whereas 0.5% GO showed accelerated mechanical deterioration, consistent with enhanced water penetration and hydrolytic sensitivity.

### 3.5. Long-Term Stability in Saline Environments

#### Hydrolytic Mass Loss Testing

**Figure 14:**
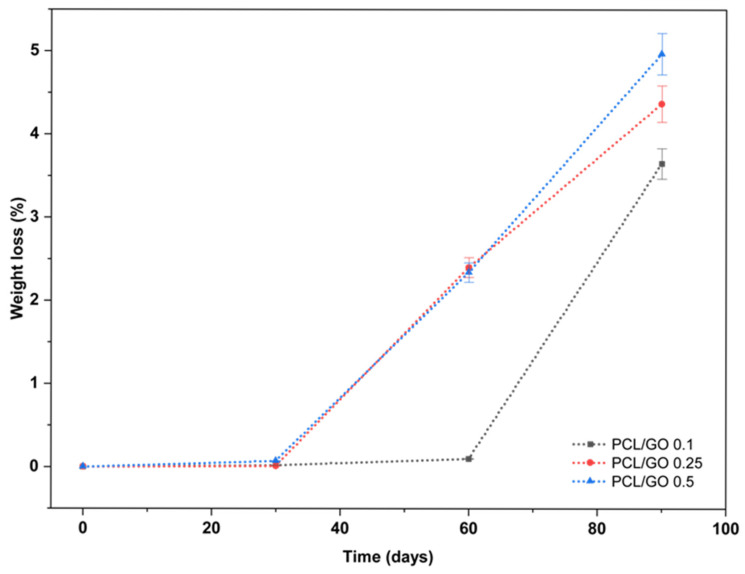
Percentage mass loss of PCL/GO films exposed to natural Ionian seawater at 15 °C for 90 days, showing the effect of GO concentration on mass change over the exposure period.

Mass loss of the samples over the course of 90 days is illustrated in Figure 14. The first 30 days demonstrated negligible degradation of all samples (mass loss < 0.1%). The curves for all GO concentrations overlap closely, suggesting that seawater penetration and ester hydrolysis were still extremely limited at low temperature. The dense structure of the films and the slow degradation kinetics of PCL in cold marine environments dominate the behavior, and the influence of GO loading is not yet apparent during this early phase.

Between 30 and 60 days, the curves begin to separate, revealing the first measurable effects of GO concentration. The PCL/GO 0.1 film reveals an almost flat response, with only 0.10% mass loss, in sharp contrast to the pure PCL film (2.81%). This behavior indicates that a small amount of GO significantly enhances film stability, likely by acting as a crystallization nucleator that increases PCL crystallinity and reduces the free volume available for water diffusion. Mass losses of PCL/GO 0.25% and 0.5% samples were similar to PCL.GO 0.1% at 30 days, indicating that at this higher loading level, GO may not uniquely form a barrier but may instead be incorporated as localized hydrophilic areas within the PCL matrix.

After 90 days, all films exhibit a clear increase in degradation, but the extent varies markedly with GO concentration. The PCL/GO 0.1% film consistently maintains the lowest degradation (3.65%), indicating its superior long-term stability. The mass loss of the 0.25% and 0.5% treatments is much steeper than that of the control, with 0.5% GO at 4.97%. With bio colonization activity, the greater mass loss indicates that increased loading of GO introduces more oxygen-containing functional groups, increases microstructural irregularities, enhances the films’ ability to absorb seawater, and increases the rate of hydrolysis of the ester linkage [71]. The influence of GO concentration on the degradation behavior of PCL in seawater agrees with previously reported findings [72,73,74,75]. The limited mass loss observed after 90 days is considered evidence of early-stage hydrolysis and possible leaching, not true biodegradation, which is characterized by longer timescales in marine settings. Without an assessment of microbial activity, the observed mass loss is conservatively attributed to non-biological hydrolytic mechanisms.

As these findings suggest, the PCL/GO 0.1% film exhibits a slower degradation rate, which is advantageous for a long-term protective layer for archaeological artifacts.

## 4. Conclusions

This study presented a preliminary assessment of modified polycaprolactone (PCL) films with graphene oxide (GO) to evaluate their behavior under saline stress conditions relevant to coastal archaeological contexts. Free-standing PCL/GO films containing 0.1, 0.25, and 0.5 wt% GO were assessed following exposure to natural Ionian seawater at 15 °C for up to 90 days, focusing on surface wettability, microstructural evolution, permeability, mechanical response, and mass loss.

The present study demonstrates that GO loading plays a critical role in governing the balance between barrier performance and hydrolytic sensitivity. The 0.1 wt% GO formulation improved barrier effectiveness by increasing diffusion tortuosity while maintaining structural integrity. In contrast, higher GO content promoted increased hydrophilicity, surface roughening, and mechanical softening due to enhanced water uptake. Observed mass loss reflects early-stage hydrolysis processes and should not be interpreted as evidence of long-term biodegradation under the applied conditions. With superior stability and enhanced barrier properties, the PCL/GO 0.1 wt% film achieved the optimal compromise in performance, supporting its potential as a temporary protective material for further investigation.

## Figures and Tables

**Table 1:** A summarized ionic composition of seawater from Crotone (Ionian coast, Italy) at a temperature of 15 °C.

Parameter	Value (gL^−1^)
pH	8.05 (unitless)
Na^+^	11.94
K^+^	0.43
Mg^2+^	1.12
Ca^2+^	0.55
Cl^−^	18.31
SO_4_^2−^	2.67

**Table 2:** The mechanical characteristics of PCL/GO films stored in natural seawater with a constant water temperature of 15 °C. Values are reported as mean ± standard deviation (*n* = 3).

Films	Time (Days)	Young’s Modulus (MPa)	Elongation at Break (%)
PCL/GO 0.1	0	284 ± 15	7.7 ± 0.7
	30	414 ± 20	4.3 ± 0.4
	60	183 ± 12	3.7 ± 0.3
	90	144 ± 10	19.7 ± 1.8
PCL/GO 0.25	0	331 ± 18	5.7 ± 0.6
	30	346 ± 17	4.3 ± 0.4
	60	163 ± 10	8.9 ± 0.9
	90	104 ± 8	5.4 ± 0.5
PCL/GO 0.5	0	309 ± 16	6.4 ± 0.6
	30	166 ± 1	4.2 ± 0.4
	60	153 ± 9	8.0 ± 0.7
	90	104 ± 9	20.7 ± 1.9

## Data Availability

The original contributions presented in this study are included in the article. Further inquiries can be directed to the corresponding author.
